# Comparative Analysis of Mitochondrial Genomes of Five Aphid Species (Hemiptera: Aphididae) and Phylogenetic Implications

**DOI:** 10.1371/journal.pone.0077511

**Published:** 2013-10-17

**Authors:** Yuan Wang, Xiao-Lei Huang, Ge-Xia Qiao

**Affiliations:** 1 Key Laboratory of Zoological Systematics and Evolution, Institute of Zoology, Chinese Academy of Sciences, Chaoyang District, Beijing, People's Republic of China; 2 College of Life Sciences, University of Chinese Academy of Sciences, Shijingshan District, Beijing, People's Republic of China; University of Veterinary Medicine Hanover, Germany

## Abstract

Insect mitochondrial genomes (mitogenomes) are of great interest in exploring molecular evolution, phylogenetics and population genetics. Only two mitogenomes have been previously released in the insect group Aphididae, which consists of about 5,000 known species including some agricultural, forestry and horticultural pests. Here we report the complete 16,317 bp mitogenome of *Cavariella salicicola* and two nearly complete mitogenomes of *Aphis glycines* and *Pterocomma pilosum*. We also present a first comparative analysis of mitochondrial genomes of aphids. Results showed that aphid mitogenomes share conserved genomic organization, nucleotide and amino acid composition, and codon usage features. All 37 genes usually present in animal mitogenomes were sequenced and annotated. The analysis of gene evolutionary rate revealed the lowest and highest rates for COI and ATP8, respectively. A unique repeat region exclusively in aphid mitogenomes, which included variable numbers of tandem repeats in a lineage-specific manner, was highlighted for the first time. This region may have a function as another origin of replication. Phylogenetic reconstructions based on protein-coding genes and the stem-loop structures of control regions confirmed a sister relationship between *Cavariella* and pterocommatines. Current evidence suggest that pterocommatines could be formally transferred into Macrosiphini. Our paper also offers methodological instructions for obtaining other Aphididae mitochondrial genomes.

## Introduction

Mitogenome is becoming a vital model for many scientific disciplines such as animal health, comparative and evolutionary genomics, molecular evolution, phylogenetics and population genetics [1]–[5]. In comparison with single mitochondrial genes widely used as markers in phylogenetics and phylogeography, the mitogenome sequences can be more phylogenetically informative, and provide multiple genome-level characteristics, such as relative position of different genes, RNA secondary structures, and modes of control of replication and transcription [2], [3], [6], [7]. Mitogenome of insects is a small double-stranded circular molecule of 14–20 kb in length and contains 37 genes including 13 protein-coding genes (PCGs), 22 transfer RNA genes (tRNA) and two ribosomal RNA genes (12S and 16S rDNA, the small and large ribosomal subunits) [8], [9]. Additionally, it contains a A+T-rich control region that plays a role in the initiation of transcription and replication [8].

Aphididae, an insect family in Sternorrhyncha of Hemiptera, consists of about 5,000 known species based on phenotypic, life cycle-specific, and host-specific variations [10], [11]. This family includes some important agricultural, forestry and horticultural pests, and distributes mainly in the temperate regions of the Northern Hemisphere and subtropical regions. Most aphid pests are from the subfamily Aphidinae, which includes the tribes Aphidini and Macrosiphini [11]. Aphidinae has traditionally been considered a sister group to the relatively small aphid group of pterocommatines (*Pterocomma*, *Plocamaphis*, etc.), which is treated as a subfamily (Pterocommatinae) in Aphididae by some aphid taxonomists [12], [13]. However, the classification status of pterocommatines has been inconclusive. It has been given a subfamilial status [12]–[14] or a tribial status in Aphidinae [15] in different classification systems. Recently, a molecular phylogenetic study has revealed that this group clustered together with *Cavariella*, a genus in Macrosiphini, and formed a sister lineage to the remaining macrosiphines [16]. This relationship between pterocommatines and *Cavariella* has been also revealed in other studies recently [17], [18]. Although this indicates that pterocommatines may be incorporated into Macrosiphini, these authors have not formally presented the change of classification. Thus, mitogenomes of *Cavariella* and pterocommatines may provide unique and indispensable information for testing this relationship.

Up to now, fifty-nine complete or nearly complete Hemipteran mitogenomes are available in GenBank (http://www.ncbi.nlm.nih.gov; accessed on November 1st, 2012), among which forty-two are from Heteroptera, and only 10 are from Sternorrhyncha. Before this study, only two complete mitogenomes of aphids, i.e. *Schizaphis graminum* [19] and *Acyrthosiphon pisum* [20], have been released. The above data analysis may indicate the relative difficulty of obtaining aphid mitogenome sequences. The difficulty may attribute to several reasons. First, it is difficult to extract high concentration DNA of aphids for their relatively small size. Some species could not even offer enough DNA with one individual. Second, the mitogenome sequences of aphids have complex secondary structure and highly A+T portion. Third, it is uneasy to amplify an entire control region which contains large tandem repetitions or complex structures. Finally, mitogenomes of aphids contain a variable repeat region which has serial tandem repetitions similarly to the control region. Other hemipteran groups may also encounter some of these difficulties [21], [22].

In the present study, one complete mitogenomes of *Cavariella salicicola*, a host-alternating species between *Salix* and Umbelliferae, and two nearly complete mitogenomes of *Aphis glycines* (Aphidini), a major and serious pest of soybean, and *Pterocomma pilosum*, a typical species with Salicaceae as primary hosts and of phylogenetic importance, were sequenced (Table 1). Together with the previously released complete mitogenomes of *S. graminum* and *A. pisum*, we compared the sequences and genome architectures of aphid mitogenomes for the first time. We also evaluated the phylogenetic positions of *C. salicicola* and *P*. *pilosum* based on sequences of protein-coding genes (PCGs).

**Table 1:** General information of the species used in this study.

Classification	Species	Mitochondrial sequence	Size (bp)	Accession number	Reference
Aphididae	*Cavariella salicicola*	complete	16,371	KC332935	This study
	*Aphis glycines*	ND4-ND3	13,002	KC840675	This study
	*Pterocomma pilosum*	ND4-ND3	12,529	KC840676	This study
	*Schizaphis graminum*	complete	15,720	NC_006158	[12]
	*Acyrthosiphon pisum*	complete	16,970	NC_011594	[13]
Phylloxeridae	*Viteus vitifoliae*	ND4-COIII	12,349	DQ021446.1	Direct submission

## Results and Discussion

### General features of aphid mitogenomes

**Figure 1:**
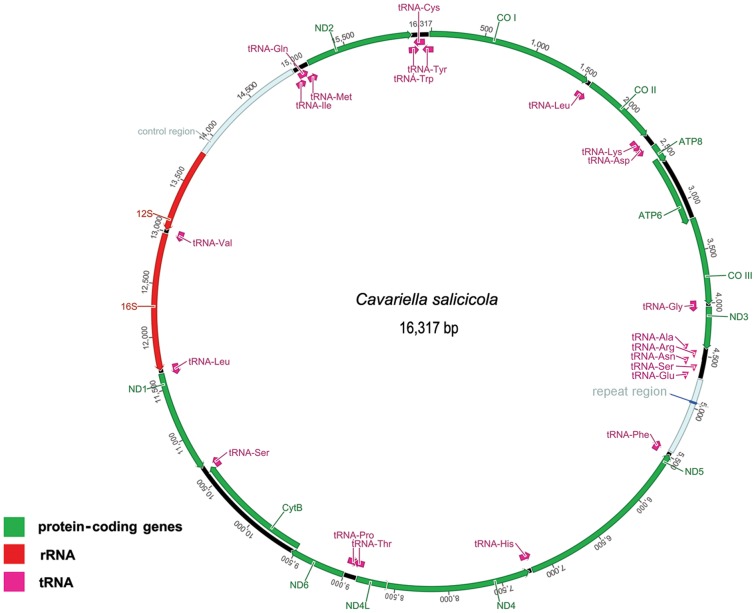
Circular map of the *Cavariella salicicola* mitogenome. Green bars represent the protein-coding genes, and grey-blue bars represent the control region and the repeat region. Red and purple bars are rRNAs and tRNAs, respectively. Arrows indicate the direction of transcription of each gene.

The complete mitogenome of *C. salicicola* with a size of 16,371 bp was obtained in the present study. Circular map of this mitogenome is shown in the Figure 1, and the organization and characteristics of it is provided in Table S1. Nearly complete mitogenomes of *A. glycines* and *P. pilosum* were obtained. Although lots of experiments were tried, the regions that we failed to amplify from these two species were usually located in or around ND3-repeat region-ND5, where extremely high A+T content, tandem repetitions, serial tRNAs and stable stem-loop structures may disrupt PCR and sequencing reactions. This is a similar problem that leads to difficulty in sequencing of other hemipteran mitogenomes [21], [22].

**Figure 2:**
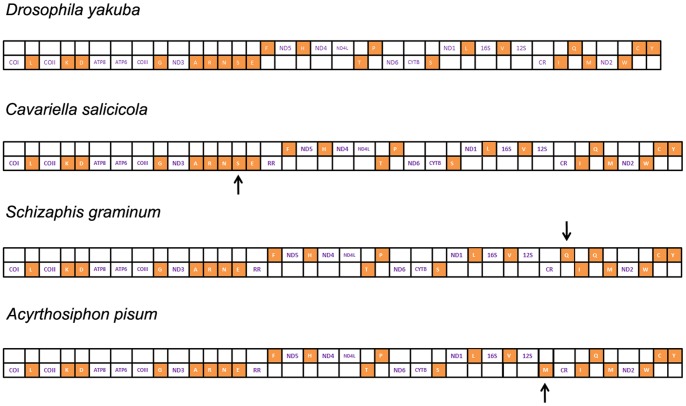
Linear comparison of complete mitogenome structure in aphids and *Drosophila yakuba*. The upper boxes indicate genes coding by N strand. Orange capital letters represent different tRNAs. Arrows refer to the tRNAs which are different from other aphid mitogenomes.

The size of *C. salicicola* mitogenome is similar to that of *S. graminum* and *A. pisum* (15,720 bp and 16,970 bp, respectively). The three complete mitogenomes were double-stranded circular molecule, except for one special repeat region, with the same gene content (37 genes and one control region) as that in the *Drosophila yakuba* (Figure 2) [23]. All genes identified were typical animal mitochondrial genes with normal gene size in the mitogenomes of Aphididae. Their length variation was minimal in PCGs, tRNAs, and the large and small rRNA subunits (12S and 16S), but obvious in the putative control regions and repeat regions. The gene order within the three complete mitogenomes was highly conserved with some tRNAs position-change exceptions: in *S. graminum* and *A. pisum*, the tRNA^Ser (AGN)^ was deleted, meanwhile, one new tRNA^Gln^ and tRNA^Met^ was inserted, respectively (Figure 2).

As the most common form of metazoan, all PCGs in the five aphid mitogenomes initiated with ATN as the start codon and mostly with TAN as the termination codon, except for COI and ND4 genes terminated with a single T residue (Table S2). The incomplete stop codon is a common phenomenon in mitogenomes of insects, and it has been proposed that the complete termination codon TAA could be generated by the post-transcriptional polyadenylation from single codon T [24], [25].

### Transfer RNAs

**Figure 3:**
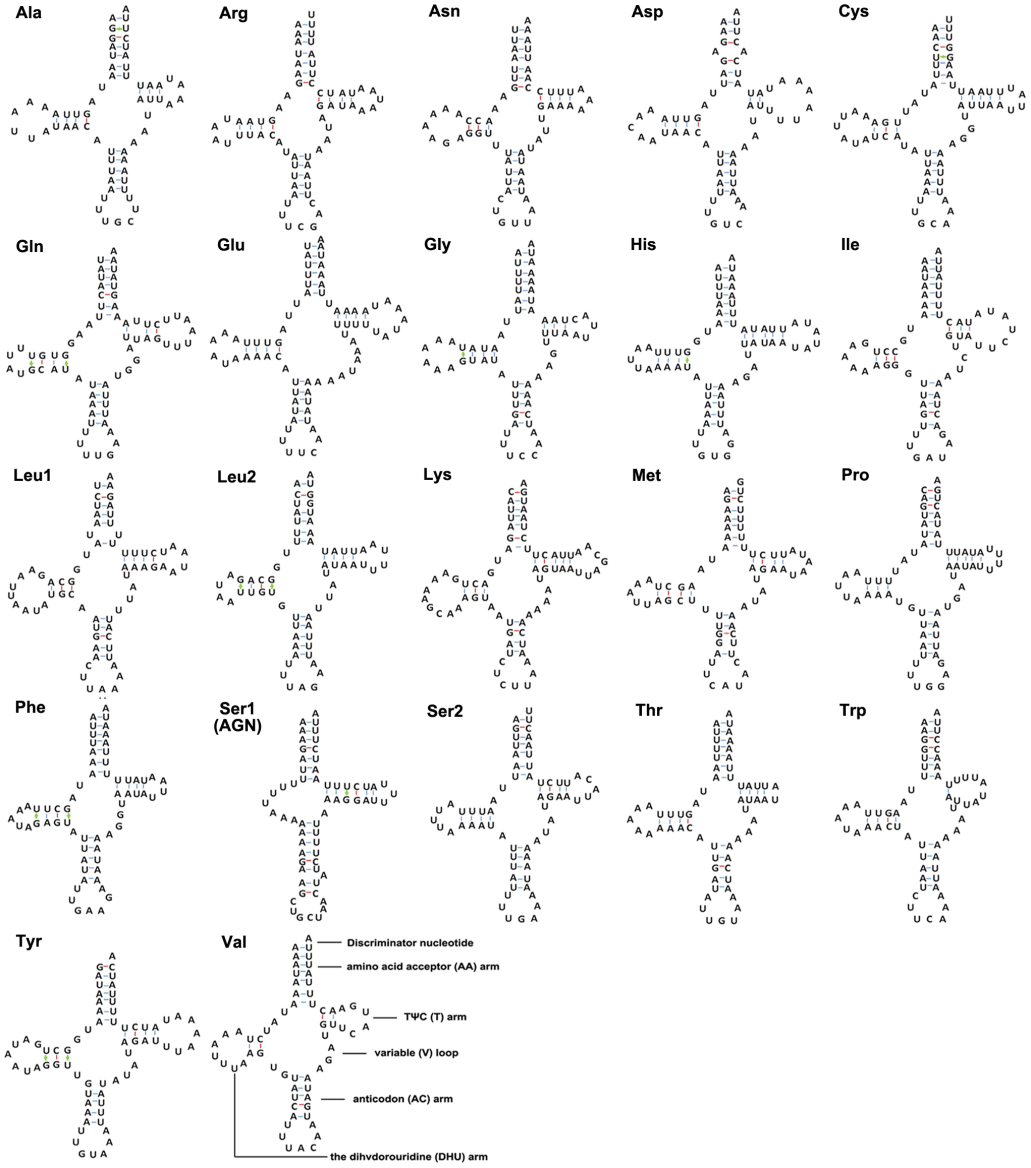
Inferred secondary structures of the 22 tRNAs in the *Cavariella salicicola* mitogenome. The tRNAs are labeled with the abbreviations of their corresponding amino acids. Dashed line (-) indicates Watson-Crick base pairing and (+) indicates G-U base pairing.

All tRNA sequences could be folded into typical cloverleaf secondary structures exposing appropriate anticodon triplets. Nearly all of them of each species represented the same structures and their sequence similarity ratios were all above 94%. Therefore, pattern for each tRNA family was modeled as reference the structure determined for *C. salicicola* (Figure 3). The whole set of 22 tRNAs typically in arthropod mitogenomes was found in *C. salicicola*. Nineteen of them were determined using tRNAscane-SE [26]. The tRNA^Arg^, tRNA^Asn^ and tRNA^Ser (AGN)^ genes were determined through comparison with previously published hemipteran mitogenomes [21], [27]. The length of tRNAs ranged from 60 to 73 bp. The aminoacyl (AA) stem (7 bp) and the AC loop (7 nucleotides) were extremely conserved, and the DHU and TΨC (T) arms were size variable mostly, within which the loop size (3–9 bp) was more variable than the stem size (2–5 bp). The size of the anticodon stems was conservative, with the exception of tRNA^Ser (AGN)^ in *C. salicicola* which possessed a long optimal base pairing (9 bp in contrast to the normal 5 bp) and a bulged nucleotide in the middle of the AC stem (Figure 3). This phenomenon is common in hemipteran mitogenomes, and has been considered as a typical feature of metazoan mtDNA [28]. However, the tRNA^Ser (AGN)^ has not been found in the other two aphids (Figure 2). This may be not an exception in arthropods considering the absence of tRNA^Gln^ in the mitogenome of the whitefly *Aleurodicus dugesii* [19], and of tRNA^Asp^ in the scorpion *Centruroides limpidus* [29]. Even in *Shinkaia crosnieri* (Decapoda: Anomura) mitogenome, tRNA^Ser (UCN)^, tRNA^Trp^, tRNA^Cys^ and tRNA^Tyr^ are all missing [30]. Deficiencies of tRNA genes are often observed in protozoans, fungi, algae, plants and low metazoans [31]. Aberrant loops, non-Watson-Crick matches, or even extremely short arms are found in some *C. salicicola* tRNA genes. It is not known whether the aberrant tRNAs lose their function, but a post-transcriptional RNA editing mechanism responsible for maintaining the function of these tRNA genes has been proposed [32], [33]. These deficiencies have been also found in some metazoan mtDNAs including several hemipteran insects [34], [35].

### Ribosomal RNAs

The characteristics of rRNA secondary structures in aphids have never been inferred before. Therefore, it is unclear how many variations exist in the rRNA structure and whether difference in length are due to the loss of helices or the reduction in helix size. The boundaries of rRNA genes were determined by sequence alignment with that of *S. graminum* [19] and *A. pisum* [20]. Like other insect mitogenomes, the large and small rRNA subunits (16S and 12S) in aphids were located at tRNA^Leu (CUN)^-tRNA^Val^ and tRNA^Val^-control region, respectively (Figure 2). The size differences in both ribosomal subunits were not distinct among different species (1, 258/9 bp in 16S and 766/7 bp in 12S). Moreover, their sequence similarity ratios were extremely high (93.5% and 93.1%). Thus, both 16S and 12S rRNAs of these species have the conserved secondary structure. This may suggest that not only the sequences but the secondary structures of rRNAs could be used in phylogenetic analyses.

**Figure 4:**
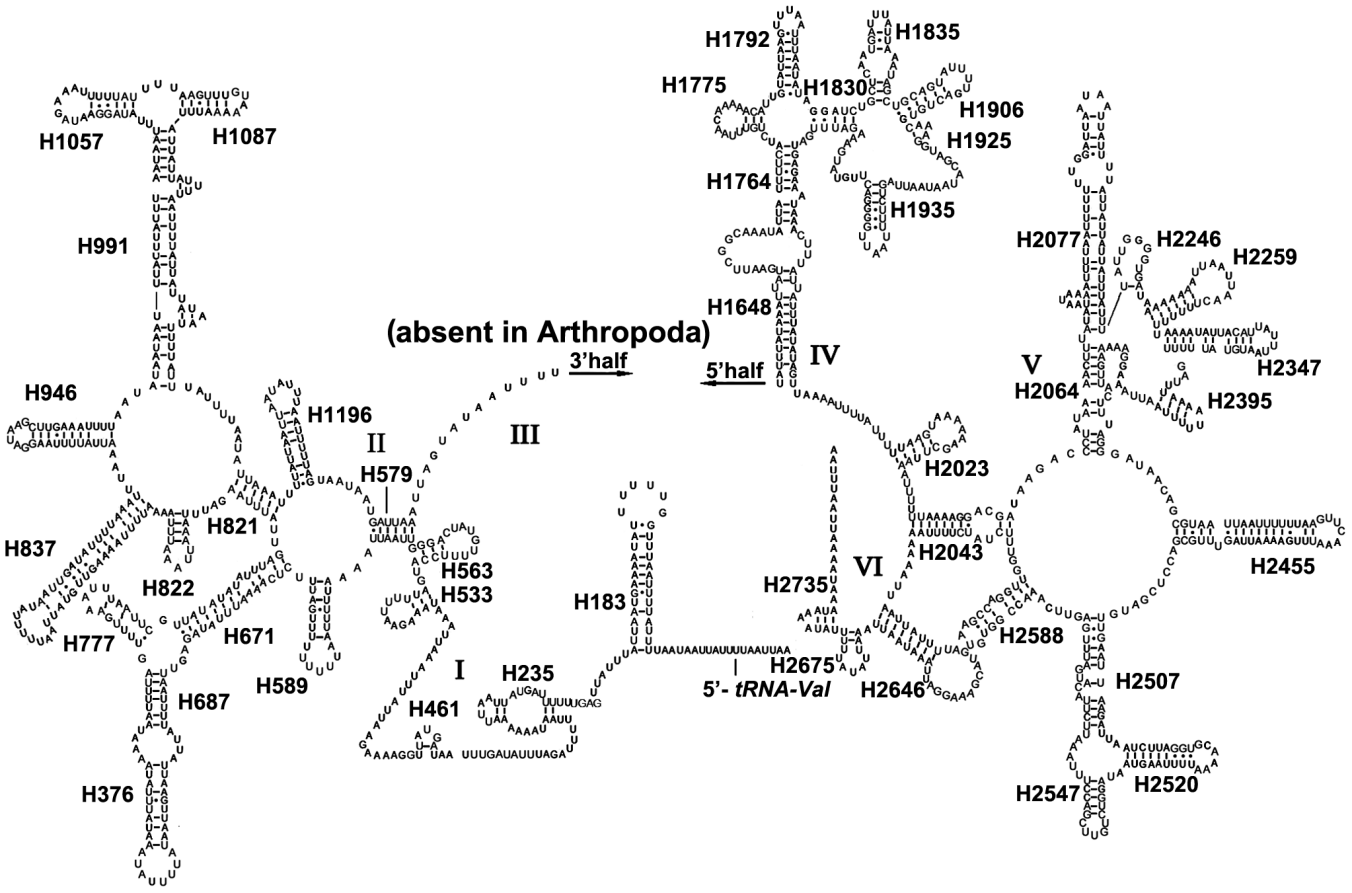
Predicted secondary structure of the 16s rRNA gene in the *Cavariella salicicola* mitogenome. Dashed (-) indicates Watson-Crick base pairing and dot (•) indicates G-U base pairing.

**Figure 5:**
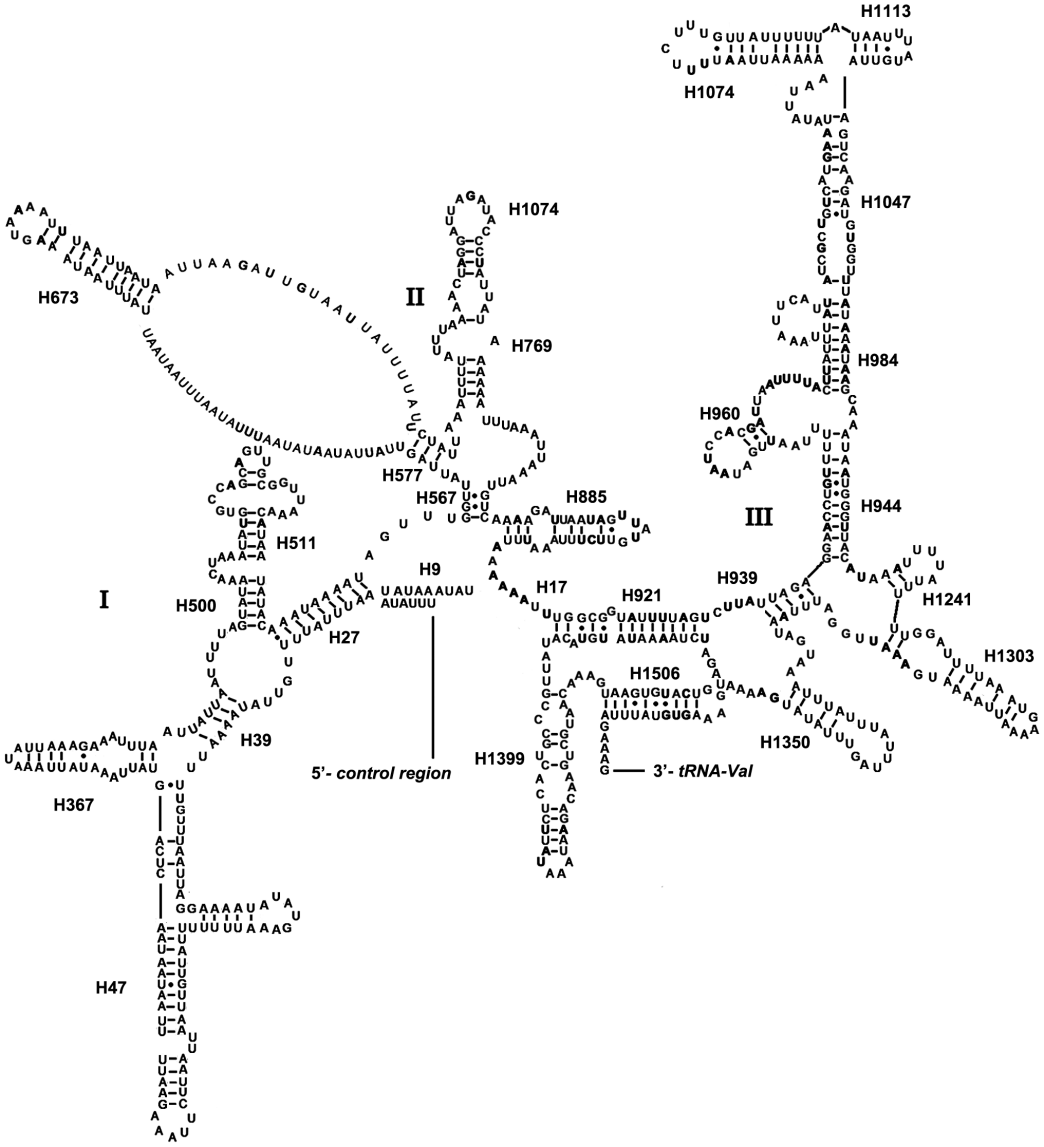
Predicted secondary structure of the 12s rRNA gene in the *Cavariella salicicola* mitogenome. Dashed (-) indicates Watson-Crick base pairing and dot (•) indicates G-U base pairing.

The rRNAs of *C. salicicola* are discussed herein as the representative of Aphididae. The secondary structure of 16S rRNA consists of six structural domains (domain III is absent in arthropods) and 45 helices (Figure 4), and the 12S rRNA consists of three structural domains and 27 helices (Figure 5). In 16S rRNA, H837 forms a long stem structure with a small loop in the terminal as observed in other insects [36], [37]. In many insect mtDNAs, the helix H2077 is absent as the bases do not form complementary pairs [37], [38], however, it includes a 17 paired bases stem and two loops in *C. salicicola*. The helix H2347 is highly variable in insects. This region in *C. salicicola* consists of 5 paired bases, which is same as that found in other hemipteran insects [24]. H2735, the last stem-loop of 16S rRNA, however, only forms a 3 bp stem and 3 bp loop in *C. salicicola*, which is different in size from similar structure of other insects [22], [37]. Domains I and II are alterable regions in terms of sequence and structure, whereas domain III is highly conserved part of the 12S rRNA of *C. salicicola*. Helix 47 is variable among different insects, but the terminal portion of this stem is conserved [25], [37]. In *C. salicicola*, two loops of Helix 47 are similar to those in *Agriosphodrus dohrni* (Hemiptera: Reduviidae) [24]. The sequences between H577 and H673 cannot be folded, similar to that in some other hemipteran insects [22], [24]. H1047 and associated stems H1068 may yield multiple possible secondary structures due to its high A+T bias and several non-canonical base pairs, as discussed in other insects [24], [37], [38].

### Nucleotide Composition

Metazoan mitogenomes usually present a clear strand bias in nucleotide composition, and the strand bias can be measured as AT- and GC-skews [39]. AT- and GC-skews of Aphididae mitogenomes are similar to the usual strand biases of metazoan mtDNA (positive AT-skew and negative GC-skew for the J-strand, whereas the reverse in the N-strand) [1]. The underlying mechanism that leads to the strand bias has been generally related to replication, which has long been assumed to be asymmetric in the mtDNA and could therefore affect the occurrence of mutations between the two strands [40]. It is possible that the overall genome A-bias is driven by mutational pressure on the N-strand and the GC-skew may be correlated with the asymmetric replication process of the mtDNA [41]. The signs of AT skew on the entire J-strand and all PCGs were reversed relative to that of the whole Aphididae mitogenome sequences (Table 2).

**Table 2:** Nucleotide composition of five aphid mitogenomes.

	Whole genome sequences			All protein-coding genes		
species	AT skew	GC skew	A+T (%)	AT skew	GC skew	A+T (%)
*Schizaphis graminum*	0.068	−0.261	83.9	−0.159	−0.036	83.2
*Acyrthosiphon pisum*	0.096	−0.288	84.7	−0.148	−0.043	83.6
*Cavariella salicicola*	0.082	−0.292	83.9	−0.151	−0.041	82.9
*Aphis glycines* *	0.048	−0.274	83.2	−0.123	−0.121	82.6
*Pterocomma pilosum* *	0.054	−0.281	82.2	−0.119	−0.137	81.7

“*” nearly complete mitogenomes.

Similar to other hemipterans [21], [22], Aphididae mitogenomes have high A+T content (Table 2),with values from 82.2% to 84.7%. Comparison of every component of the complete Aphididae mitogenomes indicates that the A+T content follows this pattern: control region > 16S rDNA > 12S rDNA > PCGs (Table S3). The control region of *S. graminum* has the same A+T content as that of *C. salicicola* but with different size (753 bp and 1137 bp, respectively), which indicates that the A+T content of control region has no correlation with its sequence length. This phenomenon has been also found in *Ruspolia dubia* (Orthoptera: Conocephalinae) containing a short A+T-rich region of 70 bp in length [42].

The nucleotide bias was also reflected in the codon usage. The codon usage *of C. salicicola* mtDNA was analyzed here. Base composition at each codon position of the concatenated 13 PCGs showed that the A+T content of the three codon positions were all above 80% (Table S4). Almost all codons were present in *C. salicicola* except for CUG, GCG, CGC and CCG, which reflects the influence of a strong compositional bias for A+T. Codon usage may be influenced by other molecular processes such as translational selection efficiency and accuracy, which apparently have a strong influence in organisms with rapid growth rates [43]–[45]. Additionally, the relative synonymous codon usage (RSCU) of NNU and NNA codons greater than 1 indicated that the third positions of the U/A have high frequency of codon usage in *C. salicicola* mitogenome (Table S5). There was a strong bias toward A+T-rich codons with the five most prevalent codons in *C. salicicola*, as in order, TTA (Leu), ATT (Ile), TTT (Phe), ATA (Met) and AAT (Asn) (Table S5).

### Gene evolutionary rate

**Figure 6:**
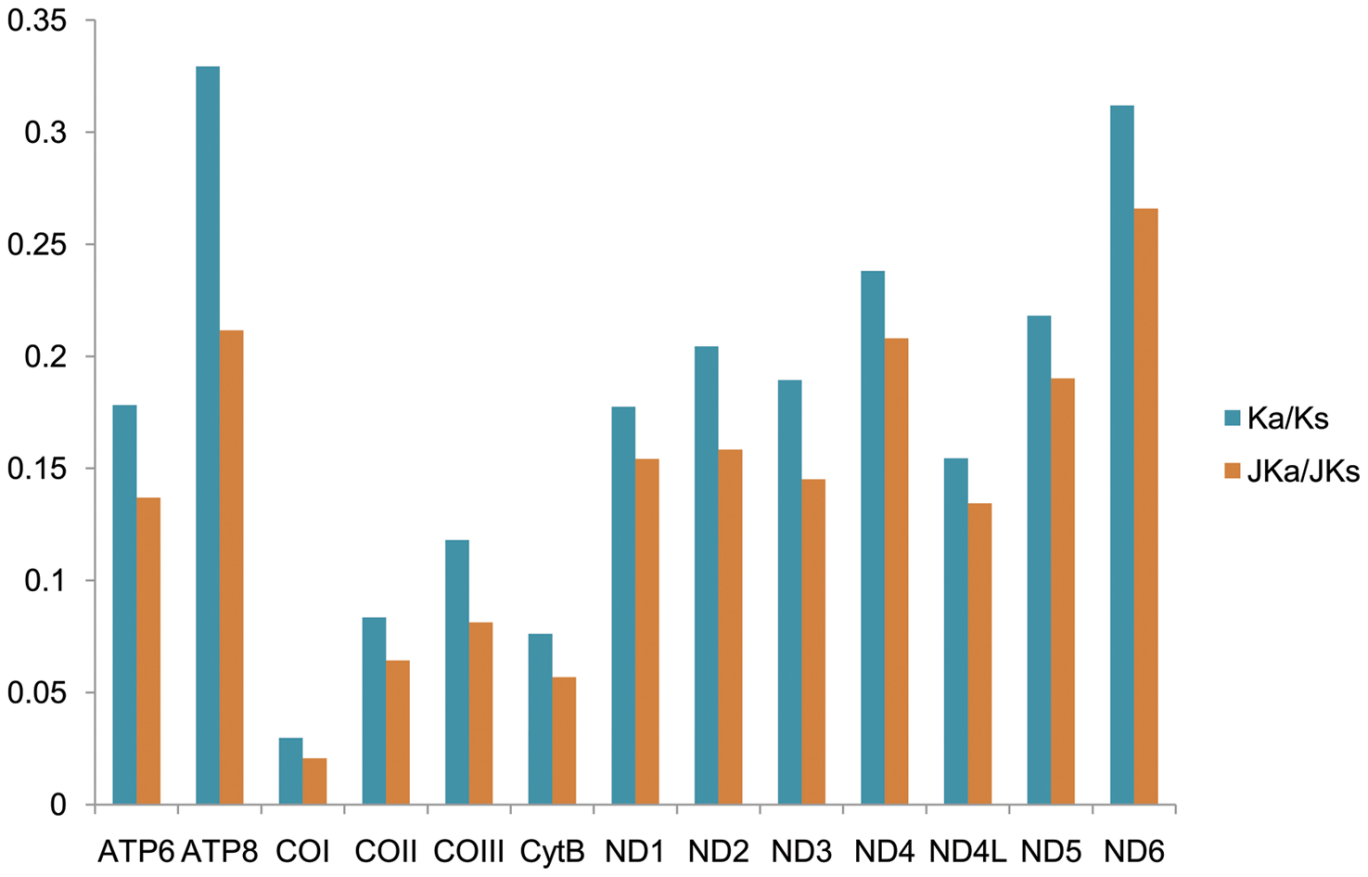
Evolutionary rates of protein-coding genes in aphid mitogenomes. Blue bar indicates the gene's Ka/Ks, and brown bar indicates the Jukes-Cantor adjusting data.

In order to analyze the gene evolutionary rate of Aphididae mitogenomes, the rates of non-synonymous substitutions (Ka, pi modified) and synonymous substitutions (Ks, pi modified) as well as the Ka/Ks ratio were calculated for all the PCGs (Figure 6). ATP8 showed the highest evolutionary rates, while COI appeared to be with the lowest rate, which coincides with the fact that COI is used as a common barcoding marker in aphids [46]. Analogously, CytB and COII with relatively slow rates may also be candidate barcoding markers. Jukes-Cantor adjusted Ka/Ks (JKa/JKs) was also calculated. Notably, the ratios of Ka/Ks and JKa/JKs for all PCGs were below 0.5, indicating that these genes were evolving under the purifying selection. Thus, we could combine all of them to analyze the phylogeny of aphids.

### Repeat region

**Figure 7:**
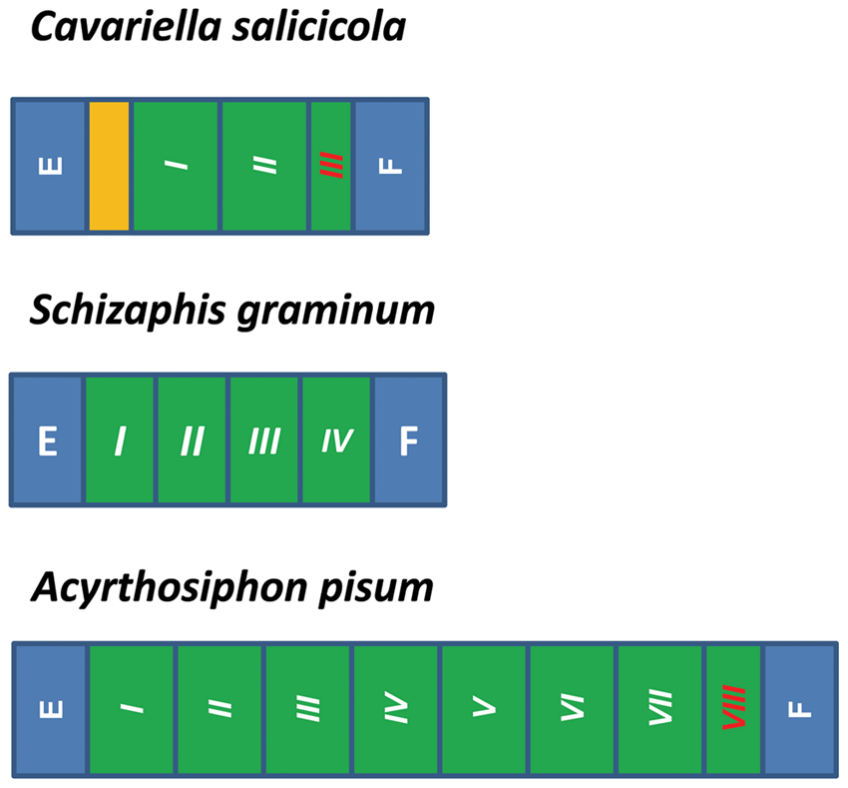
The organization of the repeat regions of aphid mitogenomes. E refers to tRNA^Glu^, and F refers to tRNA^Phe^. Green bars are the tandem repeats. Red Roman numbers refer to partial copy of the anterior portion of the repeat unit. Yellow bar is the region before the tandem repeats.

In general, the non-coding regions of insect mitogenome consist of a control region and short intergenic spacers. However, in *C. salicicola*, *S. graminum* [19] and *A. pisum*, except for a long control region and some small intergenic spacers (Table S1), a special repeat region was found between tRNA^Phe^ and tRNA^Glu^. In spite of some base sites shifts, the repeat regions of the aphid mitogenomes mainly contained a lot of tandem repeats (Figure 7). However, the tandem repeats of these three mitogenomes were different in composition, length and number of repeats. In *C. salicicola*, this repeat region included two 199-bp tandem repeats (near to tRNA^Phe^) and one 192-bp copy of the anterior portion of the repeat unit. Seven 205-bp tandem repeats with one 81-bp copy tail composed the repeat region of *A. pisum*, while *S. graminum* included four 152-bp tandem repeats.

Interestingly, this spacer cannot be found in any existing mitogenomes in GenBank. The variation of this special repeat region of different aphid mitogenomes indicates that the tandem repeats have been formatted after speciation events of these aphids, and then amplified independently. We speculate that this region full of tandem repeats has a function similar to the intergenic spacers from *Triatoma dimidiata* and *Agriosphodrus dohrni*, which are believed to be another origin of replication [19], [24], [27].

A unique region of 112-bp before the tandem repeats has been also found in *C. salicicola*, which has no similarity to other aphid mitogenome sequences and any existing sequences in GenBank. We propose two models to explain this formation. One is insertion. This 112-bp region may have originated in the mitogenome of *C. salicicola* independently by insertion after its divergence from other aphid species. The second is deletion. This spacer may have existed in the mitogenome of the ancestor of these aphid species, then was eliminated in species such as *A. pisum* and *S. graminum* but reserved in *C. salicicola*. This 112-bp region may lead the tandem repeats and influence the early replication. However, whether this region exists in mitogenomes of other aphid taxa, and its structural and functional characteristics, need to be further investigated in the future.

### Control region

**Figure 8:**
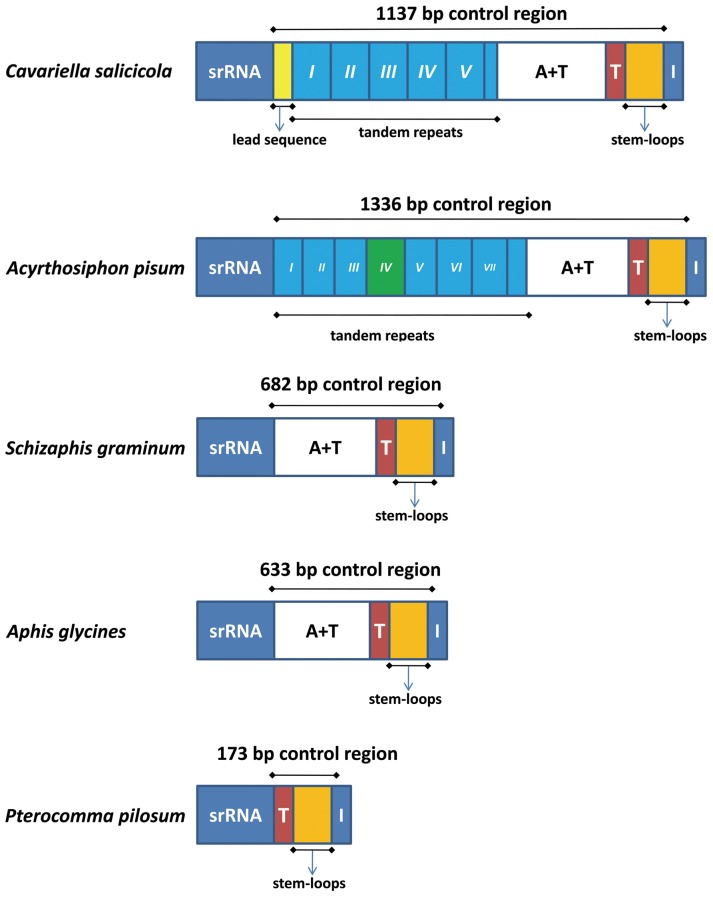
The organization of the control regions of aphid mitogenomes. The lead region is in yellow box; the blue boxes with roman numerals indicate the tandem repeat region; A+T represents high A+T content region; red boxes refer to the poly-thymidine stretch; orange boxes indicate the stem-loops region.

**Figure 9:**
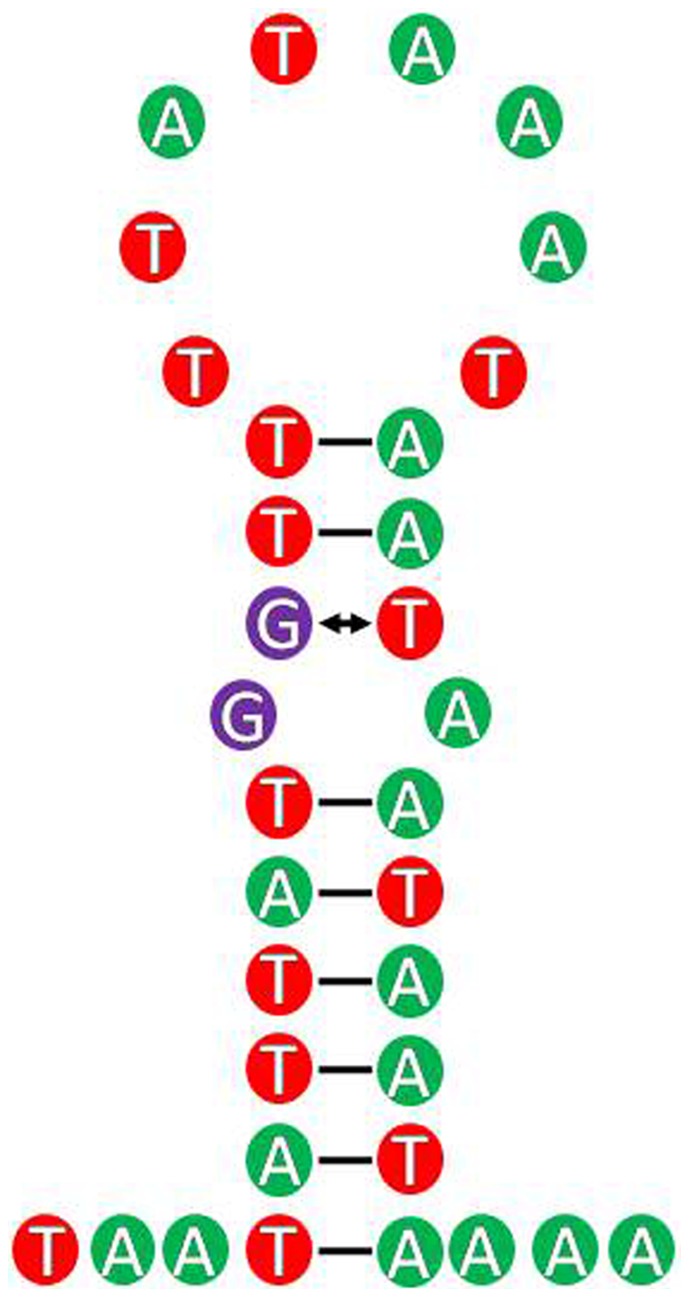
The putative secondary structure of the lead sequence of the control region in *Cavariella salicicola*. Inferred Watson-Crick bonds are illustrated by lines, GU bonds by arrows.

**Figure 10:**
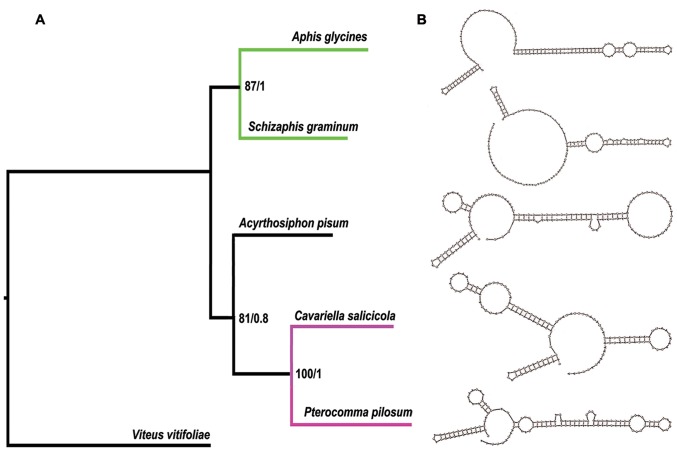
Phylogenetic tree inferred from aphid mitogenome sequences and the secondary structures of stem-loop structure regions in control regions. (A) Numbers on branches refer to Bayesian posterior probabilities followed by ML bootstrap values. Green lines refer to Aphidini and purple lines refer to Pterocommatinae + *Cavariella*. (B) Watson-Crick and GU bonds are illustrated by lines. Other noncanonical pairs are by circles.

The control regions of aphid mitogenomes are located at the conserved position between 12S rDNA and tRNA^Ile^-tRNA^Gln^-tRNA^Met^ gene cluster (Figure 2). The comparison of control region elements of all five Aphididae mitogenomes is shown in Figure 8. The control region of *C. salicicola* can be divided into five parts: a 49-bp lead region adjacent to 12S rDNA, which could fold a stem-loop structure with 34 bp (Figure 9) and may guide the mtDNA replication and transcription; a region composed of five complete tandem repeats and a partial copy of the anterior repeat unit; a 437-bp region heavily biased toward A+T (81%); a conserved poly-thymidine stretch before the stem-loop region; a stem-loop region at the end of the control region containing three potential stem-loop structures (Figure 10B). The control regions of *S. graminum*, *A. glycines* and *P. pilosum* have no many tandem repeats. However, the control region of *A. pisum* includes several parts similar to that of *C. salicicola*: a region composed of seven complete tandem repeats and a partial of the anterior repeat unit without a lead sequence region but with some sites of the fourth repeat shifted by substitution (transition or transversion); an A+T-rich region with 82.5% A+T bases; a poly-thymidine stretch and a stem-loop structure region. Only the poly-thymidine stretch and stem-loop structure region are conserved in the control regions. The stem-loop structure regions of the five aphid mitogenomes form two or three stem-loops along the mainly linear sequence (Figure 10B), which may be a widespread feature in Aphididae. Two stem-loops are present in *A. glycines* and *S. graminum*, but three are in *A. pisum*, *C. salicicola* and *P. pilosum*. This has a phylogenetic implication that pterocommatines are closer to Macrosiphini species than Aphidini. In addition, stem-loop structures in the control region have been suggested as the site of initiation of secondary strand synthesis [25], [33].

### Phylogenetic implications from mitogenome sequences

The use of protein-coding gene sequences of the mitogenomes has become an informative strategy for inferring phylogenetic relationships [47]. As groups of phylogenetic and evolutionary importance in Aphididae, the mitogenomes sequenced herein, especially that of *C. salicicola* and *P. pilosum*, can not only provide phylogenetic implications, but test the efficacy of combined PCG sequences for resolving ambiguous phylogenetic relationships.

The phylogenetic trees generated from Bayesian and maximum likelihood (ML) analyses based on 11 PCGs have similar topologies (Figure 10A). The monophyly of Aphidini was recovered with high statistical support, which is consistent with the traditional taxonomic views based on morphology [48], [49] and previous molecular phylogenetic studies [17], [50], [51]. The sister relationship between *Cavariella* and *Pterocomma* revealed by previous molecular studies [16]–[18] has been also confirmed by the phylogeny based on PCGs of mitogenomes. Pterocommatines appear to exhibit pleisiomorphic traits and have been previously considered as the sister group to Aphidinae. For example, pterocommatines are larger aphids and morphologically different from Macrosiphini species in their caudas, siphunculi, and bodies [16]. However, *Cavariella* and pterocommatines have similarity in biology. Their primary hosts are Salicaceae plants [52]; moreover, the morphological characters of fundatrices of them are almost identical to their offsprings, respectively [50], [51].

The stem-loop structures in control regions (Figure 10B) indicate structural homology in mitogenomes of *A*. *pisum*, *C. salicicola* and *P. pilosum*. This new evidence also supports a closer relationship between *Pterocomma* + *Cavariella* (P+C group) and Macrosiphini. Meanwhile, this relationship of P+C group was also proved by most phylogenetic analyses of single protein-coding genes (Figure S1, S2). Considering current evidence from our and previous molecular phylogenies, secondary structures of mitogenomes as well as biological similarity between pterocommatines and *Cavariella*, a practical and acceptable solution may be to formally treat pterocommatines as members of Macrosiphini. In the future, molecular phylogenetic studies based on more pterocommatine species and genomic data are needed to further test this proposal.

## Conclusions

The present paper reports one complete and two nearly complete mitogenomes of aphids, and provides a first comparative analysis of mitochondrial genomes of aphids. Our results suggest that gene size, gene content, and base composition are conserved among Aphididae mitogenomes. Most tRNAs can be folded as classic clover-leaf structures, with the exception of tRNA^Ser (AGN)^, in which its DHU arm simply forms a loop. Among the protein-coding genes, ATP8 represents the highest evolutionary rate, whereas COI appears to be the lowest. A special repeat region full of tandem repeats between tRNA^Phe^ and tRNA^Glu^ is found exclusively in aphid mitogenomes for the first time. We speculate that this region has a function as another origin of replication, similar to the control region. Phylogenetic reconstructions based on protein-coding genes and the stem-loop structures in control regions support a sister relationship between *Cavariella* and pterocommatines. Current evidence suggest that pterocommatines could be formally transferred into Macrosiphini.

## Materials and Methods

### Ethical treatment of animals

Aphids are invertebrates, and all species in our study are agricultural pests, which are not included in the “List of Protected Animals in China”. Ethical approval was not required for work with these aphid species. We confirm that all these species are not protected in any way, and no endangered or protected species was involved. Therefore, no specific permissions were required for field collections for this study in Gansu, Heilongjiang and Inner Mongolia.

### Samples

All species used in this study were collected in China between 2005 and 2008 (Table S6). All samples for slide-mounted specimens were stored in 70% ethanol, while specimens for molecular experiments were stored in 95% ethanol. Samples and voucher specimens were deposited in the National Zoological Museum of China at Institute of Zoology, Chinese Academy of Sciences, Beijing, China.

### Genomic DNA extraction, PCR amplification, cloning and sequencing

Samples preserved in 95% ethanol were used to extract total DNA using the DNeasy Tissue Kit (QIAGEN, Germany), according to the manufacturer's protocols. Most times, we used only one individual for DNA extraction; however, individuals from the same colony were used if one individual did not offer enough DNA.

The mitogenomes were amplified in overlapping PCR fragments (Table S7). Initially, five fragments were amplified using the universal primers from previous work with some primers modified. Other six fragments were then amplified using designed matching primers based on the read of these five fragments for the secondary PCRs. These 11 fragments covered the whole mitogenome and each of them had more than 30 bps overlap. All the primers used in this study were synthesized by Invitrogen Biotech (Beijing, China).

To obtain these total eleven fragments, short PCRs and long PCRs were used in conjunction. Short PCRs (sequence length <1.5 k) were performed with Taq DNA polymerase (TransGen Biotech, Beijing, China) as the following settings: 95°C for 3 min; 35 cycles of 92°C for 1 min, 48°C–55°C for 1 min and 72°C for 2 min. A final extension step of 10 min at 72°C was added after cycling. Long PCRs (sequence length >1.5 k) were carried out using High Fidelity (HiFi) Taq DNA polymerase (TransGen Biotech, Beijing, China) with the reaction volume recommended by the instructions under the following cycling conditions: 2 min at 94°C, 40 cycles of 30 s at 94°C, 1 min at 50–55°C, 3–6 min at 68°C depending on the size of fragments, and a final elongation step at 68°C for 15 min. The reaction conditions for some long fragments which were difficult to amplify included 1 cycle of 2 min at 92°C, 10 cycles (10 s at 92°C, 30 s at 65°C, and 4.5 min at 68°C), 20 cycles (10 s at 92°C, 30 s at 65°C, and 4.5 min at 68°C 1 cycle elongation of 30 s for each cycle), and a prolonged elongation for 7 min at 68°C.

All fragments from short PCRs were sequenced directly after purification with *EasyPure* PCR purification Kit (TransGen Biotech, Beijing, China), and all of the long PCRs products were cloned into pMD19-T sequencing vector (TaKaRa, Dalian, China). Internal primers were applied to all fragments to complete the sequences by primer walking. Sequencing reactions were performed using the corresponding PCR primers from both directions with BigDye Terminator v3.1 Cycle Sequencing Kit (Applied Biosystems, Foster City, USA) and run on an ABI 3730 automated sequencer (Applied Biosystems, USA).

### Assembling annotation, sequence analysis and inference of secondary structures

The sequence data have been deposited into GenBank (Table 1). Sequences from overlapping fragments were assembled by aligning neighboring fragments using SeqMan (DNAStar Inc., USA). Protein-coding regions and ribosomal RNA genes were identified by sequence comparison with published insect mitogenome sequences (especially the whole mitogenome of *S. graminum* NC_006158.1 and *A. pisum* NC_011594.1). The nucleotide sequences of PCGs were translated based on the invertebrate mtDNA genetic code. A+T content and codon usage were calculated using MEGA version 5.05 [53]. The putative control region was examined for regions of potential inverted repeats or palindromes by using the mfold web server (http://www.bioinfo.rpi.edu/applications/mfold/). Strand asymmetry was calculated using the formulae AT skew  =  [A−T]/[A+T] and GC skew  =  [G−C]/[G+C] [39], for the strand encoding the majority of the protein-coding genes.

The software packages DnaSP 5.0 [54] was used to calculate the synonymous substitution rate (Ks) and the nonsynonymous substitution rate (Ka) for each PCG as well as Jukes-Cantor adjusted Ka/Ks (JKa/JKs).

The tRNAs were predicted by tRNAscan-SE Search Server v.1.21 [26] with default setting. Some tRNA genes that could not be found by tRNAscan-SE were identified by comparing to other hemipterans and edited by eye. Secondary structures of the small and large subunits of rRNAs were inferred using models predicted from *Drosophila melanogaster* and *D. virilis* [55], *Ruspolia dubia* [42] and *Agriosphodrus dohrni* [24]. Stem-loops were named according to the convention of Gillespie et al. (2006) [36], as well as Cameron et al. (2008) [37].

### Phylogenetic analysis

Phylogenetic trees were reconstructed based on protein-coding gene sequences of the mitogenomes of five Aphididae species (Table 1). *V. vitifoliae* (Hemiptera: Phylloxeridae) was used as outgroup. Since the mitogenomes of *V. vitifoliae* is incomplete with only 11 PCGs without ND3 and ND5, a DNA alignment was inferred from the amino acid alignment of these 11 PCGs using MEGA version 5.05 [53], and alignments of individual genes were then concatenated excluding the stop codons.

Maximum likelihood (ML) and Bayesian inference (BI) analyses were implemented by using PHYML 3.0 [56] and MrBayes version 3.1.2 [57], respectively. ModelTest 3.7 [58] was used in combination with PAUP*4.0 [59] to select appropriate nucleotide substitution model. The GTR+I+G model was the optimal one according to the Akaike information criterion (AIC). ML analyses using PHYML were under the optimal substitution model obtained from ModelTest, and model parameter values were estimated during the analyses. Nodal support among branches was evaluated by bootstrap analysis with 100 replicates.

In the Bayesian analyses, the values of model parameters were treated as unknown variables with uniform prior probabilities, and were estimated during the analyses. One cold and three heated MCMC chains were run for one million generations, with trees sampled every 100 generations, and starting from a random tree. MCMC runs were repeated twice to test whether the chains provided valid samples from the posterior probability distribution. The first 2500 trees (25%) for each data set were discarded as burn-in, and the remaining trees were used to construct Bayesian consensus trees. An examination of the log likelihood scores and the average standard deviation of split frequencies (less than 0.01) among parallel chains suggested that the burn-in periods were long enough for chains to become stationary.

At the same time, the phylogenetic analyses of single protein-coding genes were also conducted using ML and BI approaches and the same procedures above for the 11 PCGs.

## Supporting Information

Figure S1 **Phylogenetic trees inferred from maximum likelihood analysis of single protein-coding genes.** Numbers on branches refer to ML bootstrap values.
(TIF)

Figure S2 **Phylogenetic trees inferred from Bayesian analysis of single protein-coding genes.** Numbers on branches refer to Bayesian posterior probabilities.
(TIF)

Table S1 **Organization of** ***Cavariella salicicola*** **mitogenome.**
(DOC)

Table S2 **Start and stop codons of the five aphid mitogenomes.**
(DOC)

Table S3 **Genomic characteristics of complete mitogenomes of aphids.**
(DOC)

Table S4 **Nucleotide composition of the** ***Cavariella salicicola*** **mitogenome.**
(DOC)

Table S5 **Codon usage in the mitogenome of** ***Cavariella salicicola*** **.**
(DOC)

Table S6 **Information of three sequenced Aphididae species included in the present study.**
(DOC)

Table S7 **Primers used in this study.**
(DOC)
