# Supplementary figures and images for: Comparative Analysis of Mitochondrial Genomes of Five Aphid Species (Hemiptera: Aphididae) and Phylogenetic Implications
Source: PLoS One. 2013 Oct 17;8(10):e77511. doi: 10.1371/journal.pone.0077511 (PMC3798312; doi:10.1371/journal.pone.0077511)

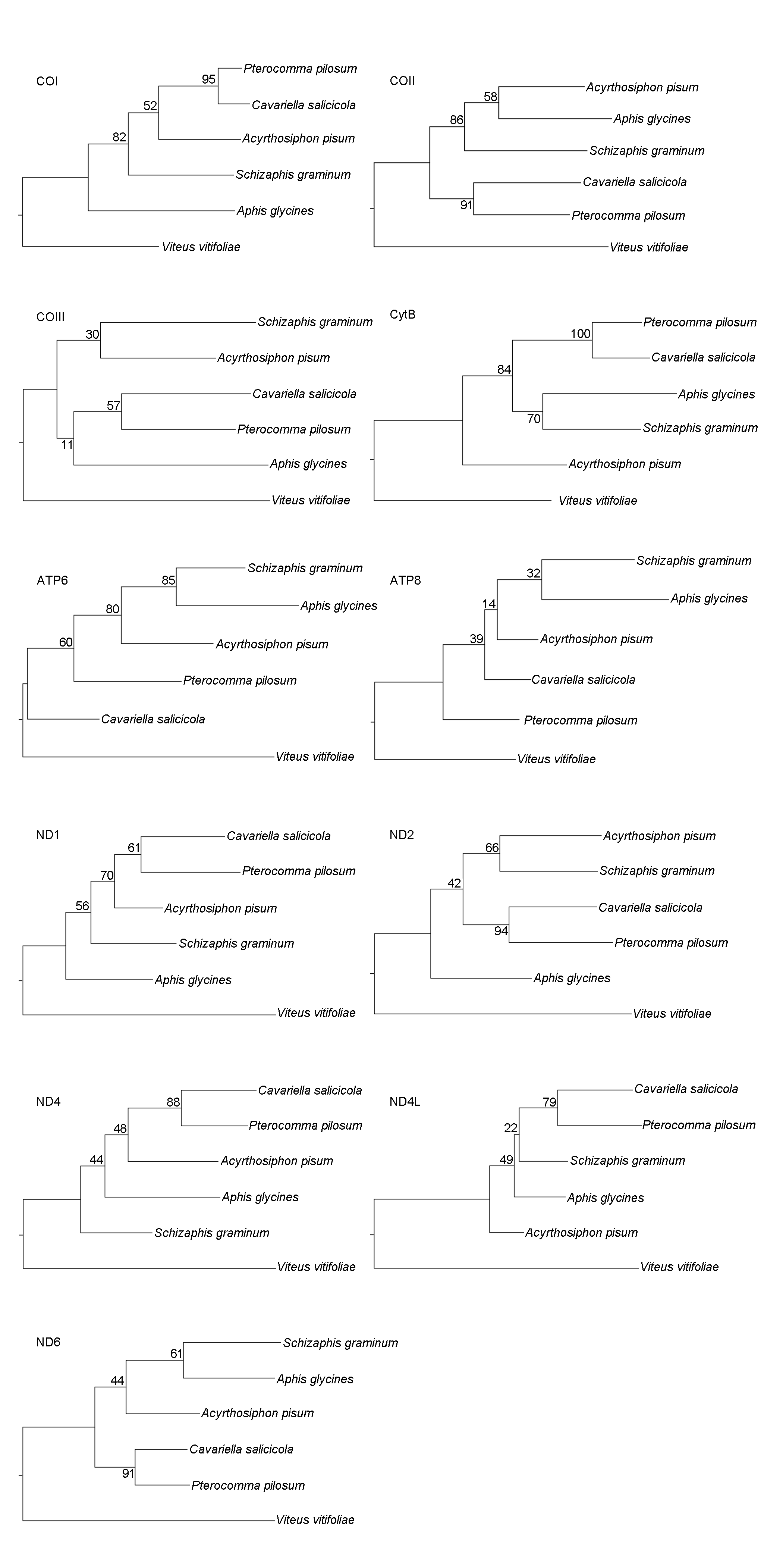

Supplement: Figure S1 — Phylogenetic trees inferred from maximum likelihood analysis of single protein-coding genes. Numbers on branches refer to ML bootstrap values. (TIF) [file pone.0077511.s001.tif]

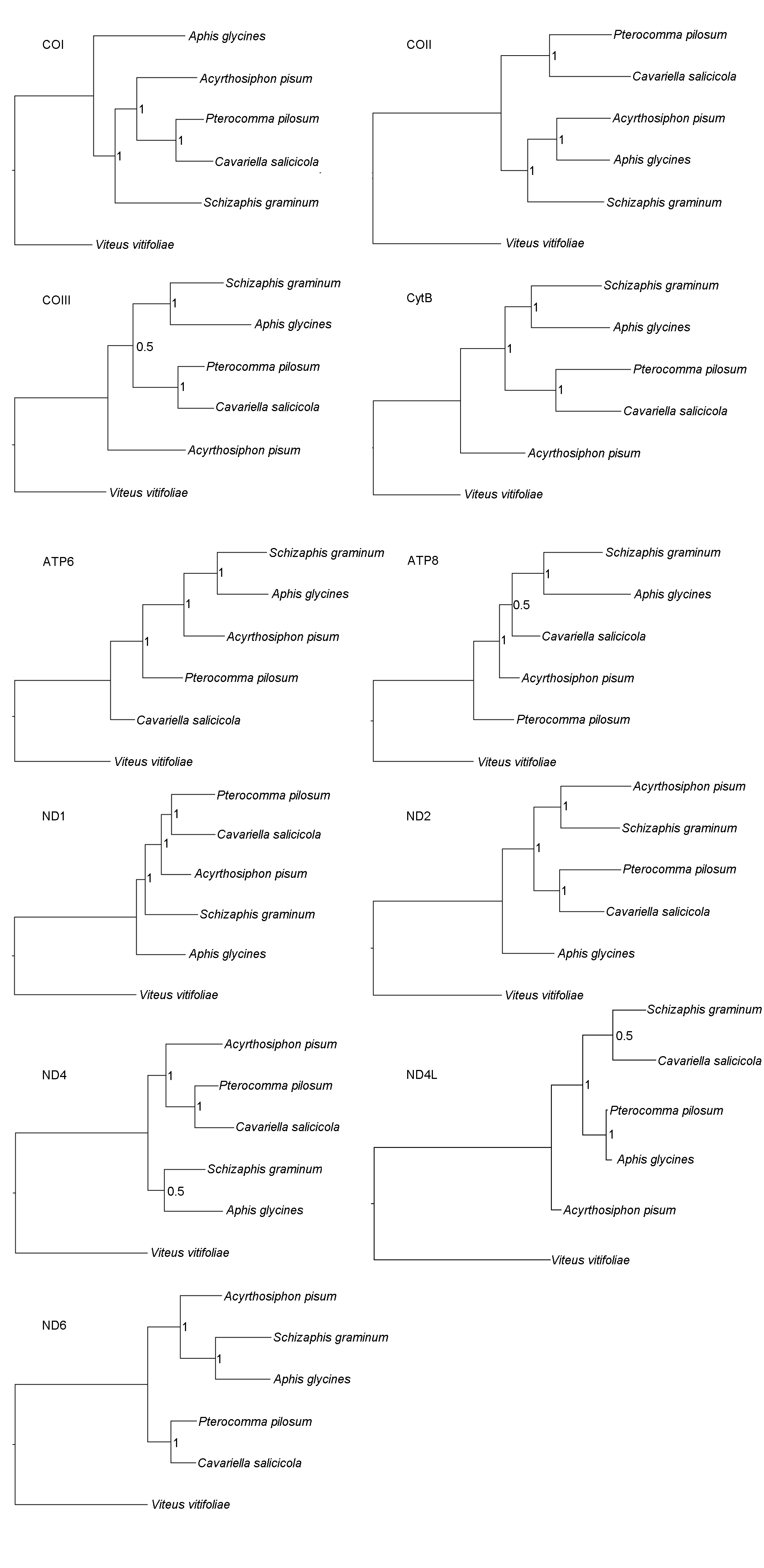

Supplement: Figure S2 — Phylogenetic trees inferred from Bayesian analysis of single protein-coding genes. Numbers on branches refer to Bayesian posterior probabilities. (TIF) [file pone.0077511.s002.tif]
