# Supplementary material for: Comparative Analysis of Mitochondrial Genomes of Five Aphid Species (Hemiptera: Aphididae) and Phylogenetic Implications
Source: PLoS One. 2013 Oct 17;8(10):e77511. doi: 10.1371/journal.pone.0077511 (PMC3798312; doi:10.1371/journal.pone.0077511)
Supplement: Table S1 — Organization of Cavariella salicicola mitogenome. (DOC) [file pone.0077511.s003.doc]

**Table S1. Organization of *Cavariella salicicola* mitogenome**

| **Gene** | **Strand** | **Position** | **Anticodon** | **Size（bp）** | **Start codon** | **Stop codon** | **Intergenic nucleotides*** |
| --- | --- | --- | --- | --- | --- | --- | --- |
| COⅠ | J | 1~1531 |  | 1531 | ATA | T |  |
| tRNA-Leu | J | 1532~1599 | TAA | 68 |  |  |  |
| COⅡ | J | 1605~2276 |  | 672 | ATA | TAA | 5 |
| tRNA-Lys | J | 2279~2351 | CTT | 73 |  |  | 2 |
| tRNA-Asp | J | 2352~2415 | GTC | 64 |  |  |  |
| Atp8 | J | 2426~2575 |  | 148 | ATA | TAA | 10 |
| Atp6 | J | 2556~3209 |  | 654 | ATA | TAA | -20 |
| COⅢ | J | 3209~3994 |  | 786 | ATG | TAA | -1 |
| tRNA-Gly | J | 3995~4058 | TCC | 64 |  |  |  |
| ND3 | J | 4056~4412 |  | 357 | ATA | TAA | -3 |
| tRNA-Ala | J | 4413~4474 | TGC | 62 |  |  |  |
| tRNA-Arg | J | 4474~4539 | TCG | 66 |  |  | -1 |
| tRNA-Asn | J | 4539~4602 | GTT | 64 |  |  | -1 |
| tRNA-Ser | J | 4602~4661 | GCT | 60 |  |  | -1 |
| tRNA-Glu | J | 4669~4739 | TTC | 71 |  |  | 7 |
| repeat region |  | 4740~5441 |  | 702 |  |  |  |
| tRNA-Phe | N | 5442~5505 | GAA | 64 |  |  |  |
| ND5 | N | 5506~7176 |  | 1671 | ATT | TAA |  |
| tRNA-His | N | 7177~7244 | GTG | 68 |  |  |  |
| ND4 | N | 7245~8553 |  | 1309 | ATA | T |  |
| ND4L | N | 8562~8852 |  | 291 | ATA | TAA | 8 |
| tRNA-Thr | J | 8854~8915 | TGT | 62 |  |  | 1 |
| tRNA-Pro | N | 8918~8983 | TGG | 66 |  |  | 2 |
| ND6 | J | 9019~9513 |  | 495 | ATT | TAA | 35 |
| CytB | J | 9513~10628 |  | 1116 | ATG | TAG | -1 |
| tRNA-Ser | J | 10627~10691 | TGA | 65 |  |  | -2 |
| ND1 | N | 10702~11637 |  | 936 | ATT | TAA | 10 |
| tRNA-Leu | N | 11638~11702 | TAG | 65 |  |  |  |
| l-rRNA | N | 11703~12960 |  | 1258 |  |  |  |
| tRNA-Val | N | 12961~13022 | TAC | 62 |  |  |  |
| s-rRNA | N | 13035~13801 |  | 767 |  |  | 12 |
| control region |  | 13802~14938 |  |  |  |  |  |
| tRNA-Ile | J | 14939~15002 | GAT | 64 |  |  |  |
| tRNA-Gln | N | 15000~15065 | TTG | 66 |  |  | -3 |
| tRNA-Met | J | 15070~15135 | CAT | 66 |  |  | 4 |
| ND2 | J | 15136~16113 |  | 978 | ATA | TAA |  |
| tRNA-Trp | J | 16114~16175 | TCA | 62 |  |  |  |
| tRNA-Cys | N | 16168~16235 | GCA | 68 |  |  | -8 |
| tRNA-Tyr | N | 16251~16317 | GTA | 67 |  |  | 15 |

* indicates the intergenic spacer, negatives indicate the nucleotide number of gene overlap.
