# Supplementary material for: Comparative Analysis of Mitochondrial Genomes of Five Aphid Species (Hemiptera: Aphididae) and Phylogenetic Implications
Source: PLoS One. 2013 Oct 17;8(10):e77511. doi: 10.1371/journal.pone.0077511 (PMC3798312; doi:10.1371/journal.pone.0077511)
Supplement: Table S2 — Start and stop codons of the five aphid mitogenomes. (DOC) [file pone.0077511.s004.doc]

**Table S2. Start and stop codons of the five aphid mitogenomes**

| **Gene** | **Start codon** | | | | | **Stop codon** | | | | |
| --- | --- | --- | --- | --- | --- | --- | --- | --- | --- | --- |
|  | ***Cavariella salicicola*** | ***Schizaphis graminum*** | ***Acyrthosiphon pisum*** | ***Aphis glycines*** | ***Pterocomma pilosum*** | ***Cavariella salicicola*** | ***Schizaphis graminum*** | ***Acyrthosiphon pisum*** | ***Aphis glycines*** | ***Pterocomma pilosum*** |
| COI | ATA | ATA | ATA | ATA | ATA | T | T | T | T | T |
| COII | ATA | ATA | ATA | ATA | ATA | TAA | TAA | TAA | TAA | TAA |
| ATP8 | ATA | ATA | ATA | ATA | ATA | TAA | TAA | TAA | TAA | TAA |
| ATP6 | ATA | ATT | ATA | ATT | ATT | TAA | TAA | TAA | TAA | TAA |
| COIII | ATG | ATG | ATG | ATG | ATG | TAA | TAA | TAA | TAA | TAA |
| ND3 | ATA | ATA | ATA | ATA | ATA | TAA | TAA | TAA | TAA | TAA |
| ND5 | ATT | ATA | ATT |  |  | TAA | TAA | TAA |  |  |
| ND4 | ATA | ATA | ATA | ATA | ATA | T | T | T | T | T |
| ND4L | ATA | ATA | ATA | ATA | ATA | TAA | TAA | TAA | TAA | TAA |
| ND6 | ATT | ATT | ATT | ATT | ATT | TAA | TAA | TAA | TAA | TAA |
| CytB | ATG | ATG | ATG | ATG | ATG | TAG | TAG | TAA | TAA | TAG |
| ND1 | ATT | ATT | ATT | ATT | ATT | TAA | TAA | TAA | TAA | TAA |
| ND2 | ATA | ATA | ATA | ATA | ATA | TAA | TAA | TAA | TAA | TAA |
