# Supplementary material for: Comparative Analysis of Mitochondrial Genomes of Five Aphid Species (Hemiptera: Aphididae) and Phylogenetic Implications
Source: PLoS One. 2013 Oct 17;8(10):e77511. doi: 10.1371/journal.pone.0077511 (PMC3798312; doi:10.1371/journal.pone.0077511)
Supplement: Table S3 — Genomic characteristics of complete mitogenomes of aphids. (DOC) [file pone.0077511.s005.doc]

**Table S3. Genomic characteristics of complete mitogenomes of aphids**

| **species** | **Whole genome** | | **13 protein-coding** | | **16s rRNA** | | **12s rRNA** | | **Control region** | |
| --- | --- | --- | --- | --- | --- | --- | --- | --- | --- | --- |
|  | **Size(bp)** | **A+T (%)** | **No. of condons** | **A+T (%)** | **Size(bp)** | **A+T (%)** | **Size(bp)** | **A+T (%)** | **Size(bp)** | **A+T (%)** |
| *Schizaphis graminum* | 15720 | 83.9 | 3655 | 83.3 | 1259 | 85.4 | 766 | 84.1 | 753 | 85.5 |
| *Acyrthosiphon pisum* | 16970 | 84.7 | 3646 | 83.7 | 1259 | 85.0 | 767 | 83.9 | 1334 | 89.9 |
| *Cavariella salicicola* | 16371 | 83.9 | 3650 | 82.9 | 1258 | 85.0 | 767 | 83.6 | 1137 | 85.5 |
| ***Drosophila yakuba*** | **16019** | **78.6** | **3727** | **76.7** | **1326** | **83.4** | **789** | **79.3** | **1077** | **92.9** |
