# Supplementary material for: Comparative Analysis of Mitochondrial Genomes of Five Aphid Species (Hemiptera: Aphididae) and Phylogenetic Implications
Source: PLoS One. 2013 Oct 17;8(10):e77511. doi: 10.1371/journal.pone.0077511 (PMC3798312; doi:10.1371/journal.pone.0077511)
Supplement: Table S4 — Nucleotide composition of the Cavariella salicicola mitogenome. (DOC) [file pone.0077511.s006.doc]

**Table S4. Nucleotide composition of the *Cavariella salicicola* mitogenome**

|  | **A%** | **G%** | **C%** | **T%** | **A+T%** | **AT-skew** | **GC-skew** | **No. of nucleotides** |
| --- | --- | --- | --- | --- | --- | --- | --- | --- |
| Whole genome | 45.4 | 5.7 | 10.4 | 38.5 | 83.9 | 0.082 | -0.292 | 16317 |
| Protein-coding genes | 35.2 | 8.2 | 8.9 | 47.7 | 82.9 | -0.151 | -0.041 | 10946 |
| First codon | 33.5 | 7.4 | 7.9 | 51.2 | 84.7 | -0.209 | -0.033 | 3649 |
| Second codon | 39 | 8.2 | 8.6 | 44.3 | 83.3 | -0.064 | -0.024 | 3649 |
| Third codon | 33.2 | 9 | 10.1 | 47.7 | 80.9 | -0.179 | -0.058 | 3648 |
| Protein-coding genes-J | 38.8 | 6.8 | 11.3 | 43.1 | 81.9 | -0.053 | -0.249 | 6739 |
| First codon | 46.6 | 4.1 | 8.1 | 41.2 | 87.8 | 0.062 | -0.328 | 2247 |
| Second codon | 40.2 | 9.8 | 12.4 | 37.6 | 77.8 | 0.033 | -0.117 | 2246 |
| Third codon | 29.6 | 6.5 | 13.5 | 50.4 | 80 | -0.260 | -0.350 | 2246 |
| Protein-coding genes-N | 29.5 | 10.4 | 4.9 | 55.2 | 84.7 | -0.303 | 0.359 | 4207 |
| First codon | 33 | 10 | 3.8 | 53.2 | 86.2 | -0.234 | 0.449 | 1403 |
| Second codon | 27.7 | 13.6 | 6.3 | 52.4 | 80.1 | -0.308 | 0.367 | 1402 |
| Third codon | 27.7 | 7.5 | 4.8 | 60 | 87.7 | -0.368 | 0.220 | 1402 |
| tRNA genes | 45.5 | 6 | 8.6 | 39.9 | 85.4 | 0.066 | -0.178 | 1377 |
| tRNA genes-J | 46.2 | 7.2 | 7.6 | 38.9 | 85.1 | 0.086 | -0.027 | 917 |
| tRNA genes-N | 43.9 | 3.5 | 10.7 | 42 | 85.9 | 0.022 | -0.507 | 460 |
| rRNA genes | 46.2 | 4.9 | 10.6 | 38.3 | 84.5 | 0.093 | -0.368 | 2025 |
| Control region | 42.3 | 4.9 | 9.6 | 43.2 | 85.5 | -0.011 | -0.324 | 1137 |
