# Supplementary material for: Comparative Analysis of Mitochondrial Genomes of Five Aphid Species (Hemiptera: Aphididae) and Phylogenetic Implications
Source: PLoS One. 2013 Oct 17;8(10):e77511. doi: 10.1371/journal.pone.0077511 (PMC3798312; doi:10.1371/journal.pone.0077511)
Supplement: Table S5 — Codon usage in the mitogenome of Cavariella salicicola . (DOC) [file pone.0077511.s007.doc]

**Table S5. Codon usage in the mitogenome of *Cavariella salicicola***

| **AA** | **Codon** | **Number** | **RSCU** | **AA** | **Codon** | **Number** | **RSCU** |
| --- | --- | --- | --- | --- | --- | --- | --- |
| Phe(F) | UUU | 440 | 1.86 | Tyr(Y) | UAU | 151 | 1.74 |
|  | UUC | 33 | 0.14 |  | UAC | 23 | 0.26 |
| Leu(L2) | UUA | 457 | 5.06 | His(H) | CAU | 45 | 1.67 |
|  | UUG | 23 | 0.25 |  | CAC | 9 | 0.33 |
| Leu(L1) | CUU | 24 | 0.27 | Gln(Q) | CAA | 44 | 1.87 |
|  | CUC | 2 | 0.02 |  | CAG | 3 | 0.13 |
|  | CUA | 36 | 0.40 | Asn(N) | AAU | 274 | 1.86 |
|  | CUG | 0 | 0.00 |  | AAC | 21 | 0.14 |
| Ile(I) | AUU | 451 | 1.72 | Lys(K) | AAA | 128 | 1.83 |
|  | AUC | 41 | 0.16 |  | AAG | 12 | 0.17 |
| Met(M) | AUA | 294 | 1.12 | Asp(D) | GAU | 46 | 1.74 |
|  | AUG | 22 | 1.00 |  | GAC | 7 | 0.26 |
| Val(V) | GUU | 52 | 2.51 | Glu(E) | GAA | 64 | 1.80 |
|  | GUC | 2 | 0.10 |  | GAG | 7 | 0.20 |
|  | GUA | 26 | 1.25 | Cys(C) | UGU | 32 | 1.94 |
|  | GUG | 3 | 0.14 |  | UGC | 1 | 0.06 |
| Ser(S2) | UCU | 79 | 2.08 | Trp(W) | UGG | 5 | 1.00 |
|  | UCC | 7 | 0.18 |  | UGA | 80 | 3.00 |
|  | UCA | 105 | 2.76 | Arg(R) | CGU | 17 | 1.03 |
|  | UCG | 1 | 0.03 |  | CGC | 0 | 0.00 |
| Ser(S1) | AGU | 35 | 0.92 |  | CGA | 23 | 1.39 |
|  | AGC | 1 | 0.03 |  | CGG | 1 | 0.06 |
|  | AGA | 55 | 3.33 | Pro(P) | CCU | 58 | 2.11 |
|  | AGG | 3 | 0.18 |  | CCC | 6 | 0.22 |
| Thr(T) | ACU | 46 | 1.55 |  | CCA | 46 | 1.67 |
|  | ACC | 3 | 0.10 |  | CCG | 0 | 0.00 |
|  | ACA | 69 | 2.32 | Gly(G) | GGU | 66 | 1.94 |
|  | ACG | 1 | 0.03 |  | GGC | 1 | 0.03 |
| Ala(A) | GCU | 34 | 1.84 |  | GGA | 64 | 1.88 |
|  | GCC | 5 | 0.27 |  | GGG | 5 | 0.15 |
|  | GCA | 35 | 1.89 | stop | UAA | 0 | 0.00 |
|  | GCG | 0 | 0.00 |  | UAG | 0 | 0.00 |

A total of 3624 codons from *Cavariella salicicola* are analyzed, excluding the start and stop codons.

AA, amino acid; RSCU, Relative synonymous codon usage.
