# Supplementary material for: Comparative Analysis of Mitochondrial Genomes of Five Aphid Species (Hemiptera: Aphididae) and Phylogenetic Implications
Source: PLoS One. 2013 Oct 17;8(10):e77511. doi: 10.1371/journal.pone.0077511 (PMC3798312; doi:10.1371/journal.pone.0077511)
Supplement: Table S6 — Information of three sequenced Aphididae species included in the present study. (DOC) [file pone.0077511.s008.doc]

Table S6 Information of three sequenced Aphididae species included in the present study

| **Species** | **Locality** | **Time** | **Voucher** |
| --- | --- | --- | --- |
| *Cavariella salicicola* | Sunan, Gansu (38°86' N, 99°57' E) | 2007-8-16 | IOZ-20711 |
| *Aphis glycines* | Harbin, Heilongjiang (45°46' N, 125°40' E) | 2008-7-24 | SBA-01412 |
| *Pterocomma pilosum* | Yakeshi, Inner Mongolia (49°04' N, 120°10' E) | 2005-8-16 | IOZ-17911 |
