# Supplementary material for: Comparative Analysis of Mitochondrial Genomes of Five Aphid Species (Hemiptera: Aphididae) and Phylogenetic Implications
Source: PLoS One. 2013 Oct 17;8(10):e77511. doi: 10.1371/journal.pone.0077511 (PMC3798312; doi:10.1371/journal.pone.0077511)
Supplement: Table S7 — Primers used in this study. (DOC) [file pone.0077511.s009.doc]

**Table S7**. Primers used in this study

| **No. fragment** | **Mainly locus** | **Primer ID** | **Nucleotide sequence (5’-3’)** | **Reference** |
| --- | --- | --- | --- | --- |
| 1 | Co I & Co II | Lep F  A3772 | ATTCAACCAATCATAAAGATATTGG  GAGACCATTACTTGCTTTCAGTCATCT | Foottit et al., 2008  Normark et al., 1996 |
| 2 | Co II & Co III | CO2Af (mtDNA)  CO3WWRD (mtDNA) | AATCAYAGWTTTATRCCWATTCA  TCWCGAATWACATCWCGTCATCA | Ortiz-Rivas et al., 2009 |
| 3 | Co II & Co III | Co2f  Co3r | TAGATGACTGAAAAGCAAAG  GTAATCCATGAAATCCTGTT | Independent design |
| 4 | Co III & ND3 | CO3WWRDre  nad3r | TGATGACGWGATGTWATTCGWGA  GATAGTTAGCAGCTTCTTTT | Independent design |
| 5 | ND3 & ND5 | N3Jx  nad5r | CCATTTGAATGTGGWTTTGATCC  TACTTCTTTGTCCGCATCC | Simon et al., 2006, modified  Independent design |
| 6 | ND3 & ND5 | nd31  nd51 | TGAATGTGGATTTGATCCATT  CCTTTTTCTTCTTGGTTWCCT | Independent design |
| 7 | ND5 & ND4 | N5-J7572  N4-N8727 | AAAGGGAATTTGAGCTCTTTTWGT  AAATCTTTRATTGCTTATTCWTC | Simon et al., 2006 |
| 8 | ND4L & CytB | N4-J8641x  CB-N10608 | CCAGAAGAACATAAACCATG CCAAGTARTGAWCCAAARTTTCA | Simon et al., 2006, modified |
| 9 | CytB & ND1 & 16S & 12S | CP1  12sai | GATGATGAAATTTTGGATC  AAACTAGGATTAGATACCCTATTAT | Harry et al., 1996  Simon et al., 1994 |
| 10 | 12S & control region & ND2 | 16Sa  ND2/CO IFre | ATGTTTTTGATAAACAGGCG  GCAAATAATATTCATGTGGAG | Simon et al., 1994  Independent design |
| 11 | ND2& COI | ND2/CO IF  ND2/CO IR | CTCCACATGAATATTATTTGC TTCCTGATCAAATACCAAATA | Independent design |
